# Virtual Screening and Molecular Dynamics Study of Potential Negative Allosteric Modulators of mGluR1 from Chinese Herbs

**DOI:** 10.3390/molecules200712769

**Published:** 2015-07-15

**Authors:** Ludi Jiang, Xianbao Zhang, Xi Chen, Yusu He, Liansheng Qiao, Yanling Zhang, Gongyu Li, Yuhong Xiang

**Affiliations:** 1 Key Laboratory of TCM-Information Engineer of State Administration of TCM, School of Chinese Pharmacy, Beijing University of Chinese Medicine, Beijing 100102, China; E-Mails: jiangludi_52@163.com (L.J.); wzhangxb@126.com (X.Z.); chenxi_cx95@163.com (X.C.); heyusue@163.com (Y.H.); b20100222012@163.com (L.Q.); lidoc2727@163.com (G.L.); 2 Department of Chemistry, Capital Normal University, Beijing 100048, China; E-Mail: cnuxiangyh@163.com

**Keywords:** mGluR1, NAMs, virtual, pharmacophore, docking, MD, TCM

## Abstract

The metabotropic glutamate subtype 1 (mGluR1), a member of the metabotropic glutamate receptors, is a therapeutic target for neurological disorders. However, due to the lower subtype selectivity of mGluR1 orthosteric compounds, a new targeted strategy, known as allosteric modulators research, is needed for the treatment of mGluR1-related diseases. Recently, the structure of the seven-transmembrane domain (7TMD) of mGluR1 has been solved, which reveals the binding site of allosteric modulators and provides an opportunity for future subtype-selectivity drug design. In this study, a series of computer-aided drug design methods were utilized to discover potential mGluR1 negative allosteric modulators (NAMs). Pharmacophore models were constructed based on three different structure types of mGluR1 NAMs. After validation using the built-in parameters and test set, the optimal pharmacophore model of each structure type was selected and utilized as a query to screen the Traditional Chinese Medicine Database (TCMD). Then, three different hit lists of compounds were obtained. Molecular docking was used based on the latest crystal structure of mGluR1-7TMD to further filter these hits. As a compound with high QFIT and LibDock Score was preferred, a total of 30 compounds were retained. MD simulation was utilized to confirm the stability of potential compounds binding. From the computational results, thesinine-4ʹ-*O*-β-d-glucoside, nigrolineaxanthone-P and nodakenin might exhibit negative allosteric moderating effects on mGluR1. This paper indicates the applicability of molecular simulation technologies for discovering potential natural mGluR1 NAMs from Chinese herbs.

## 1. Introduction

G protein-coupled receptors (GPCRs) are seven transmembrane proteins, which contain the largest class of drug targets and almost take part in every physiological process in the human body. According to their functional similarity and sequence homology, GPCRs can be divided into six classes: class A, B, C, D, E and F [1,2]. The metabotropic glutamate receptors (mGluRs), which belong to Class C GPCRs, are expressed in neuronal and glial cells widely, and divided into three groups (I–III) based on their sequence similarity, pharmacological profiles and transduction mechanisms [3]. mGluR1, a member of the group I mGluRs, is considered as a promising therapeutic target as well as mGluR5 to treat diseases including depression, anxiety, chronic pain and Alzheimer’s disease [4].

Initially, drug development efforts of mGluRs have focused on developing candidate compounds that act at the orthosteric site. However, high failure rates occurred and it was attributed to a lack of receptor subtype selectivity derived from the highly conserved orthosteric binding site. For example, the key residues in the orthosteric binding sites of mGluR1 and mGluR5 are 100% conserved, suggesting that subtype selectivity would be very difficult to achieve [5]. This problem can be overcome by developing allosteric modulators that act on alternative binding sites. These modulators binding predominantly within the 7TMD of the class C receptors can alter the affinity or efficacy of native ligands in positive, negative, or neutral ways, which results in a spectrum of activity that cannot be achieved by orthosteric ligands alone [6]. In recent years, computer molecular simulation technologies, such as pharmacophore modeling and homology modeling, have been used to identify modulators of mGluR1 [7,8,9,10]. However, there are still some limitations in that the information derived from both the receptor structure and the receptor-ligand interactions is insufficient in terms of ligand-based virtual screening; in addition, the sequence similarity between the mGluRs and the other released GPCRs is less than 20% [11], which implies that there will be a great deal of difference between the homology model of mGluR1 and the actual structure. Fortunately, the crystal structure of the human mGluR1 7TMD bound to a NAM was reported by Wu *et al*., which was the first crystal structure of 7TMD of class C GPCRs [12]. This structure may provide a basic model for all Class C GPCRs and mGluRs drugs with better properties or novel scaffolds will be designed or discovered more successfully [13]. For example, the three dimensional structure of mGluR5 was constructed based on this structure and then novel mGluR5 PAMs were successfully found [14,15].

The purpose of this study was to screen potential NAMs of mGluR1 from TCMD (Version 2009) by using a series of molecular simulation method. It has been reported that, some ingredients of Chinese medicine produce the healing efficacy though the allosteric way. For instance, tetrandrine can exert allosteric effect by targeting on calcium channels [16], magnolol and honokiol act through an allosteric site in GABA_A_ to treat anxiety and convulsions [17]. In addition, Chinese herbs have been widely used to treat various nervous system diseases with good effect [18,19]. Thus, mGluR1 NAMs are likely to be discovered from Chinese herbs. In this study, a combination of ligand- and structure-based methods were utilized to screen mGluR1 NAMs, including pharmacophore modeling, molecular docking and molecular dynamics (MD) simulations. It is worth mentioning that previous research has shown that subtle structural changes to allosteric ligands of mGluRs result in unexpected changes in their pharmacology, such as changing NAM into positive allosteric modulator (PAM) [20]. In this case, in order to increase the specificity of virtual screening, ligand-based pharmacophore models aiming at different structure types of mGluR1 NAMs were built. After validated by the built-in parameters and test set, the best hypothesis for each structure type served as template to search potential mGluR1 NAMs from the TCMD. Moreover, the crystal structure of human mGluR1-7TMD (PDB ID: 4OR2) in the RCSB Protein Data Bank was utilized for the first time in this study to perform molecular docking [21]. With the complementary three-dimensional structure information and the receptor-ligand interaction information, the false positive rate of database screening should be decreased. MD simulations were employed to examine the stabilizing interactions between potential mGluR1 NAMs and 4OR2. Finally, three potential compounds which can be used in future mGluR1 NAMs design were obtained.

## 2. Results and Discussion

### 2.1. Pharmacophore Model Studies

Seventy four mGluR1 NAMs collected from the Allosteric Database can be classified into three groups (Group A, Group B and Group C) based on their different structure types. The number of compounds in each group is 12, 16 and 46, respectively.

**Figure 1:**
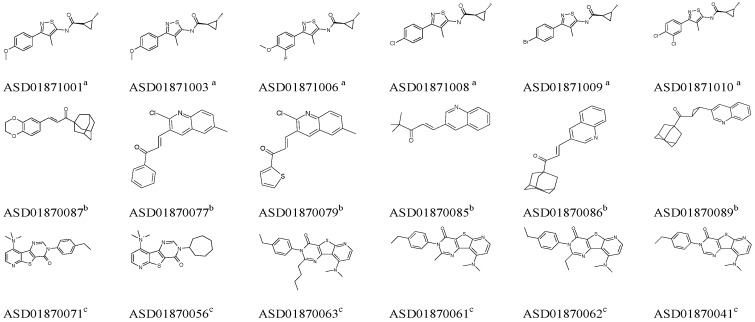
Structures of six high active mGluR1 NAMs derived from each group. The superscripts a, b and c represent the corresponding Groups A, B and C. Each six mGluR1 NAMs represented a specific chemical structure type and were defined as the training set to build an exclusive GALAHAD model.

Then, six high active molecules from each group were selected as training set compounds to construct an exclusive GALAHAD model for each group. During this process, the maximum number of pharmacophore hypotheses to generate was set up to 20 for each group. Chemical information from the training sets is shown in Figure 1.

The generated models were firstly evaluated using the built-in parameters and the top five models of each group were selected based on two criteria: (1) Number of “hits” should be equal or near to the number of active molecules, which in this case was six. (2) Smaller value of Energy and higher value of Specificity were desired for the best model [22]. Thus, 74 active compounds and 222 inactive compounds formed a test set to validate the selected pharmacophore models constructed for three structure types. Initially, the evaluation results of the selected pharmacophore models were analyzed comprehensively, but none of hypotheses possessed eligible A% and CAI (Table 1), which indicates that these models cannot identify active compounds and exclude inactive compounds well. As a result, it was difficult for each model to cover the whole pharmacophore features of three types of mGluR1 NAMs.

**Table 1:** The pharmacophore model validation results of each group.

Model	Specificity	Energy	N_Hits	Ha ^a^	Ht ^b^	Ht-Ha ^c^	A% ^d^	N ^e^	CAI ^f^
**Model_A018**	**4.33**	**2.89**	**6**	**12**	**29**	**17**	**16.22%**	**1.66**	**0.27**
Model_A014	3.97	3.57	6	58	154	96	78.38%	1.51	1.18
Model_A010	3.96	3.80	6	20	124	104	27.02%	0.65	0.17
Model_A009	3.97	3.97	6	57	141	84	77.02%	1.62	1.25
Model_A002	3.97	4.06	6	57	148	91	77.02%	1.54	1.19
**Model_B014**	**3.81**	**1.76**	**5**	**11**	**13**	**2**	**14.87%**	**3.39**	**0.50**
Model_B013	3.81	2.20	6	10	13	3	13.52%	3.08	0.42
Model_B008	3.81	1.01	6	14	31	17	18.92%	1.81	0.34
Model_B015	3.66	3.33	6	11	20	9	14.87%	2.20	0.33
Model_B006	3.81	2.01	4	14	48	34	18.92%	1.17	0.22
**Model_C002**	**4.02**	**13.66**	**4**	**39**	**41**	**2**	**52.70%**	**3.81**	**2.00**
Model_C001	4.02	19.73	4	39	42	3	52.70%	3.71	1.96
Model_C003	4.02	13.87	4	39	44	5	52.70%	3.55	1.87
Model_C004	4.02	12.78	4	39	45	6	52.70%	3.47	1.83
Model_C010	4.02	14.99	4	40	46	6	54.05%	3.48	1.88

^a^ Ha is the number of active hits using pharmacophores to search. ^b^ Ht is the number of hits using pharmacophores to search. ^c^ Ht-Ha represents the number of false positive hits using pharmacophores to search. ^d^ A% represents the ability to identify active compounds from the test database. ^e^ N represents the ability to identify active compounds from inactive compounds. ^f^ CAI is the Comprehensive Appraisal Index.

However, the goal of pharmacophore model generation is to construct an exclusive hypothesis model of each group to gather a certain structure type of active compounds rather than build a single model with low specificity to hit three different types of compounds. Thus, Model_A018, Model_B014 and Model_C002 achieved the expected goal were discovered. To be specific, Model_A018 with the smallest value of Energy and the highest value of Specificity could hit all 12 active compounds of Group A and only 17 inactive compounds in the test set, whereas, other models which hit too many inactive compounds to collect active compounds with the strucuture type of Group A were rejected. Also, the Specificity and Energy of Model_B014 as well as Model_C002 were all reasonable. Eleven active compounds of Group B were well mapped with Model_B014, while only two inactive compounds were hit by Model_B014. Similarly, 39 active compounds of Group C were filtered by Model_C002, whereas only two inactive compounds were hit. In addition, 41 mGluR1 PAMs were used as external test set to further evaluate the identification ability of the three optimal models, two PAMs mapped with model_C002, and none PAM could map model_A018 and model_B014. Therefore, these three pharmacophore models have better specificity and can distinguish NAMs from PAMs efficiently. To sum up, Model_A018, Model_B014, and Model_C002 were chosen as the best pharmacophore models to further screen the TCMD, respectively.

**Figure 2:**
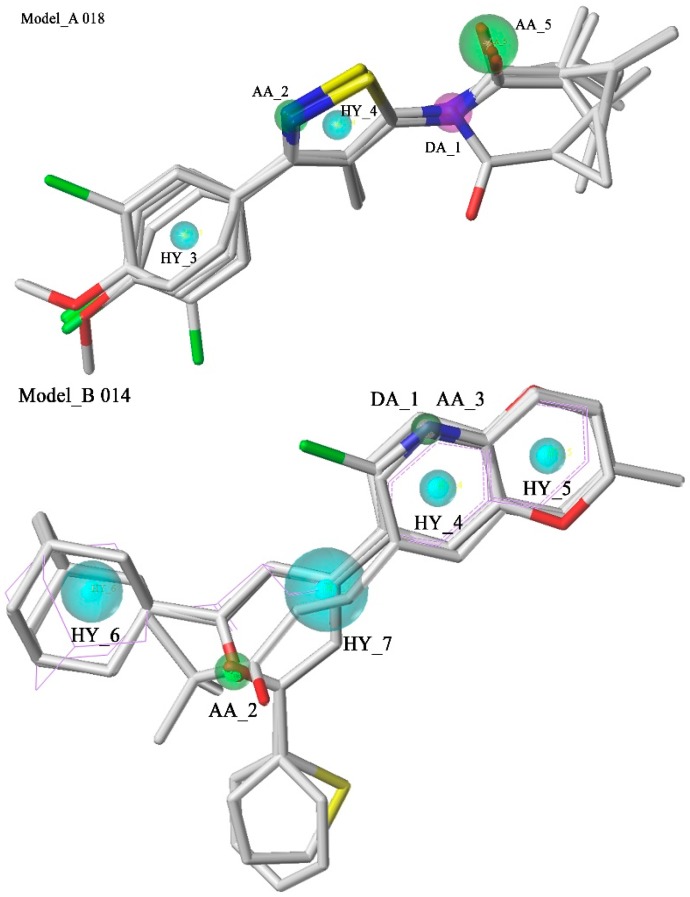

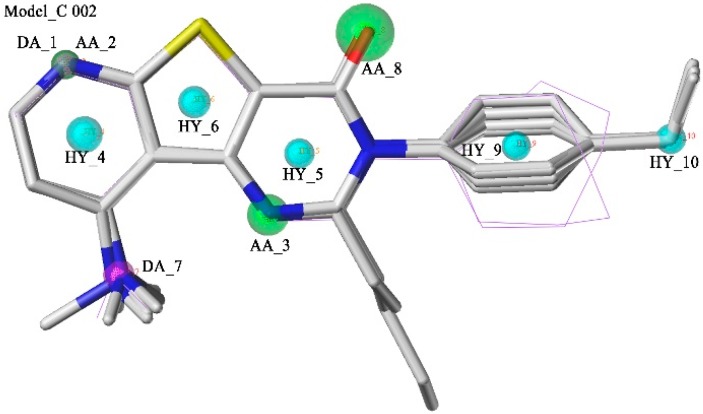
Pharmacophore models and molecular alignment of the compounds used to elaborate the model. In order, they are Model_A018, Model_B014, and Model_C002, respectively. Cyan indicates hydrophobic features, green indicates hydrogen bond acceptors, and purple indicates hydrogen bond donors.

The selected pharmacophore models are shown in Figure 2. Model_A018 consisted of five features, including one hydrogen bond donor (DA_1), two hydrogen bond acceptors (AA_2, AA_5) and two hydrophobic features (HY_3, HY_4). Model_B014 consisted of seven features, including one hydrogen bond donor (DA_1), two hydrogen bond acceptors (AA_2, AA_3) and four hydrophobic features (HY_4, HY_5, HY_6, HY_7). Model_C002 consisted of ten features, including two hydrogen bond donors (DA_1, DA_7), three hydrogen bond acceptors (AA_2, AA_3, AA_8) and five hydrophobic features (HY_4, HY_5, HY_6, HY_9, HY_10). The compounds in the training set which were not well mapped with the optimal model were marked with purple lines. In Model_B014 and Model_C002, a green sphere covered a purple sphere because the acceptor atom and the donor atom are in the same position in this compound.

### 2.2. Database Searching

The screening of the three pharmacophore queries yielded a total of 4042 hits that met the specific requirements. A query fit value (QFIT) was computed for each hit to rank the matching rate of its required structural features to the pharmacophoric query, so a high QFIT score corresponds to a good alignment between pharmacophore model and compound conformer [23]. However, choosing all these natural compounds for the next study was not a wise strategy, as only parts of compounds in the TCMD were drug-like. Therefore, in the first step of drug discovery it was necessary to apply some drug-like filters to eliminate the non-drug-like molecules and then only focus on drug-like molecules. The hits were then subjected to drug-likeness filters. In this study, one violation was tolerated when using Lipinski’s rule, so as to retain as many potential lead compounds as possible. Thus, a total of 642 potential drug-like mGluR1 NAMs satisfied four rules of Lipinski’s rule of five, including 256 hits by Model_A018, 289 filtered by Model_B014 and 97 obtained by Model_C002. Further screening of the filtered hits were carried out using the docking algorithm in DS.

### 2.3. Molecular Docking and Database Search

**Figure 3:**
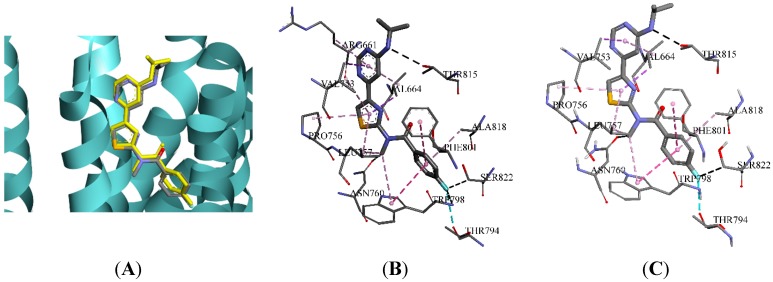
The comparison between the initial binding pose interactions and the docked pose interactions of FITM in crystal structure 4OR2_A. (**A**) The pose of FITM initially binding to 4OR2_A was compared with that of the one predicted by molecular docking (yellow); (**B**) The initial binding pose interactions in the allosteric binding site; (**C**) The docked pose interactions.

4OR2 is a homodimer structure of mGluR1. The binding pocket A was created with a sphere radius of 14.60 Å around the initial compound (FITM) present in 4OR2_A, and the radius of pocket B was 14.70 Å in 4OR2_B. Three docking algorithms in DS, LibDock, CDOCKER, and Flexible Docking, were used to evaluate their applicability of pocket A and B. The scores of initial compounds and the RMSD values between the re-docked FITM and the crystal structure are listed in Table 2. The smaller the RMSD value the better, so 4OR2_A with LibDock, which produced the smallest RMSD value of 0.56 Å (<2.00 Å), was the optimal combination [24]. Besides, the LibDock Score of FITM was 143.730, which was set to be the threshold in identifying mGluR1 NAMs by molecular docking. The comparisions between the initial binding pose interactions and the docked pose interactions of FITM in 4OR2_A are presented in Figure 3. As Figure 3A shows, there is a favorable superimposition between the binding pose and the docked pose. Besides, according to Figure 3B,C, the interactions of the binding pose were similar to that of the docked one. To be specific, both poses can form hydrogen bond interactions with THR794, SER822 and THR815, and form hydrophobic interactions with PRO756, LEU757and TRP798. The only difference is that the initial binding pose can also form a hydrophobic interaction with ARG661, however, there is no experimental support with respect to mutations confirming that interaction with ARG661 is crucial to activity. Therefore, it can be concluded that the docked pose of FITM predicted by LibDock was consistent with the binding pose.

**Table 2:** The corresponding score and RMSD value of FITM with three docking algorithms.

Indexes		4OR2_A	4OR2_B
**LibDock**	RMSD	**0.56 Å**	3.89 Å
	LibDock Score	**143.73**	146.22
CDOCKER	RMSD	1.18 Å	1.12 Å
	-CDOCKER_ENERGY	40.80	43.90
	-CDOCKER_INTERACTION_ENERGY	55.74	58.46
Flexible Docking	RMSD	1.17 Å	1.21 Å
	-CDOCKER_ENERGY	44.15	38.56
	-CDOCKER_INTERACTION_ENERGY	58.36	55.79
	LibDock Score	127.83	139.35

**Figure 4:**
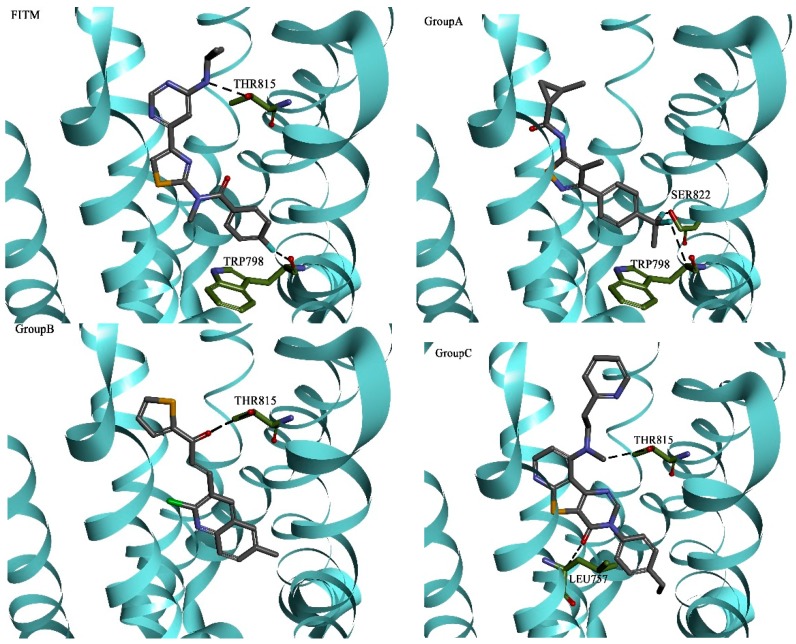
Docking results between FITM, three active NAMs from three groups and 4OR2_A.

Active NAMs of three groups were docked into the binding site A, respectively. The three-dimensional binding poses and the corresponding docking schematic diagrams of FITM and three experimental active compounds which have higher LibDock Score in different groups are illustrated in Figure 4. They all fit tightly into the same long and narrow binding pocket. The binding modes and key residues of the experimental NAMs for human mGluR1 were further analyzed. Key residues, namely high-frequency amino acid residues, are listed in Table 3. The binding mode of the active NAMs in three groups were similar to each other. Moreover, all these active NAMs can form hydrogen bonds with LEU757, THR815 and hydrophobic interactions with VAL664, LEU757, VAL753, and PRO756. Thus it can be seen that, even though the mGluR1 NAMs have different structures, they form similar interactions with the crystal structure of mGluR1.

Hydrogen bonds and hydrophobic contacts were the main ligand-receptor interactions and this was consistent with the GALAHAD pharmacophore modeling results. Moreover, the key interactions between active compounds and mGluR1 predicted by the docking study were also congruent with the mutagenesis studies. Fukuda suggested that the modulation effects of mGluR1 NAMs were significantly affected in point mutations of ASN760, TRP798, PHE801, TYR805 and THR815 [25]. They also confirmed that the THR815 mutation attenuated activities of all active compounds although the shift of the modulatory potencies of compounds was varied [25,26]. In addition, residue LEU757, the only residue in the active site that differs between the rat and human receptors, plays an important role in the activity of active compounds [27]. Thus, the above results confirmed that the approaches used in this study were rational and reliable.

**Table 3:** Key interaction and the crucial residues between mGluR1 NAMs and 4OR2_A.

	**Hydrogen Bonding Interactions**					**Hydrophobic Interactions**				
Group A	TRP798	LEU757	ARG661	THR815	THR794	VAL664	LEU757	PHE801	VAL753	PRO756
Group B	LEU757	TRP798	GLY665	ARG661	THR815	VAL664	VAL753	LEU757	ALA818	PRO756
Group C	ARG661	GLY752	THR815	LEU757	ASN760	VAL664	VAL753	PRO756	LEU757	ARG661

Next, 642 drug-like compounds were docked into the mGluR1 protein, to select the compounds on the basis of their ability to form favorable interactions with the active site. Thus 127 potential compounds which got a higher score than FITM were retained. Taking the QFIT into consideration, the top ten compounds in each group with high-scoring function values were retained for further study. Among the 30 potential compounds, thesinine-4ʹ-*O*-β-d-glucoside, nigrolineaxanthone-P and nodakenin, which had a higher QFIT and LibDock Score according to the database search results and similar interaction modes with mGluR1 to active NAMs, were regarded as possible novel mGlur1 NAM lead candidates.

**Figure 5:**
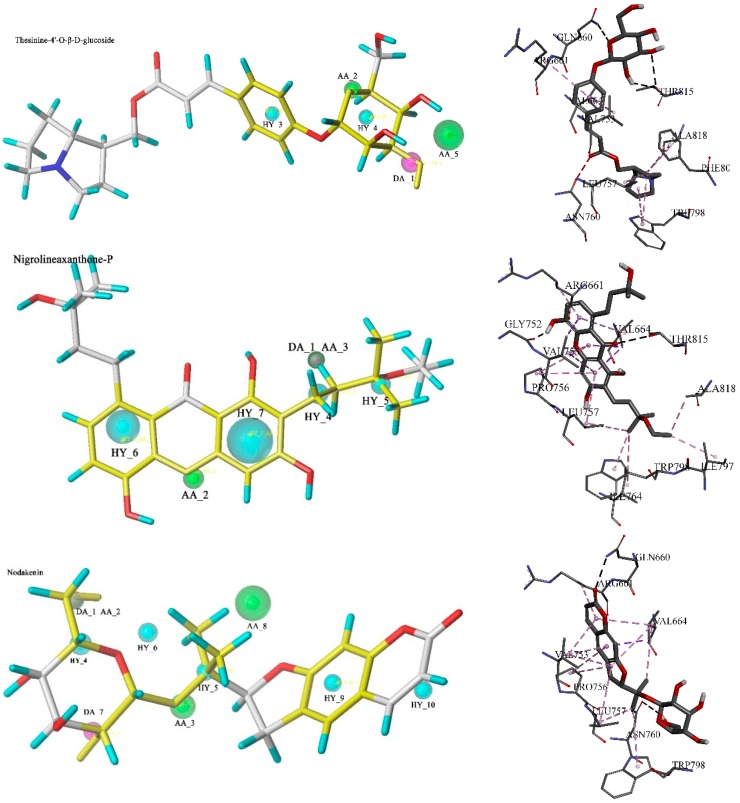
Pharmacophore mapping results and molecular docking results of thesinine-4ʹ-*O*-β-d-glucoside, nigrolineaxanthone-P and nodakenin.

Thesinine-4ʹ-*O*-β-d-glucoside mapped four features with pharmacophore model_A018 and the QFIT was 30.91, while AA_5 was unmatched. This potential compound got the highest LibDock Score, 174.93, and formed a hydrogen bond network at the mGluR1 active site with the amino acids THR815 and CYS746, also formed hydrophobic interactions with TRP798, LEU757, and ARG661 and so on, which were consistent with experimental active NAMs. In addition, the atoms which mapped with DA_1 and AA_2 also formed hydrogen bonds with THR815 and GLN660, respectively. Nigrolineaxanthone-P, which scored a QFIT of 24.86 and a LibDock Score of 170.09, formed a strong hydrogen bond interaction with THR815 and hydrophobic interactions with ILE764, TRP798, and LEU757. In the case of the pharmacophore model_B014 mapping result, DA_1 and AA_3 were not mapped, while other features were almost the same as the non-bond interactions. Nodakenin scored a QFIT of 45.73 and a LibDock Score of 153.22, and formed a strong hydrogen bond interaction with GLN660 and hydrophobic interactions with ILE661, TRP798, and LEU757. HY_6, AA_8 and HY_10 were not mapped by nodakenin. The chemical groups within this compound which formed a hydrophobic interaction with 4OR2 were also mapped with the corresponding hydrophobic features. Thus it can be seen that these three hits satisfied the expected interactions as defined by the pharmacophore models and their binding modes were similar to the initial compound in the crystal structure. Furthermore, the results of pharmacophore model were almost consistent with that of molecular docking. The three hits mapped with the corresponding pharmacophore models and the interactions with 4OR2 are shown in Figure 5.

### 2.4. Molecular Dynamics Simulation

**Figure 6:**
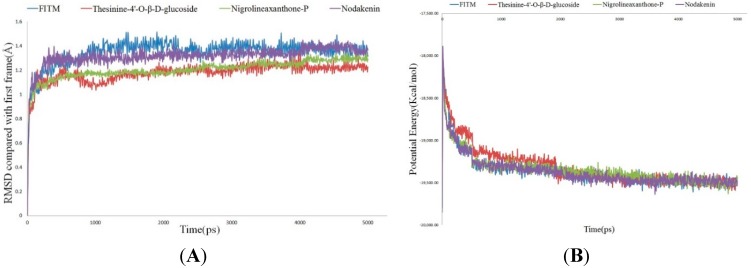

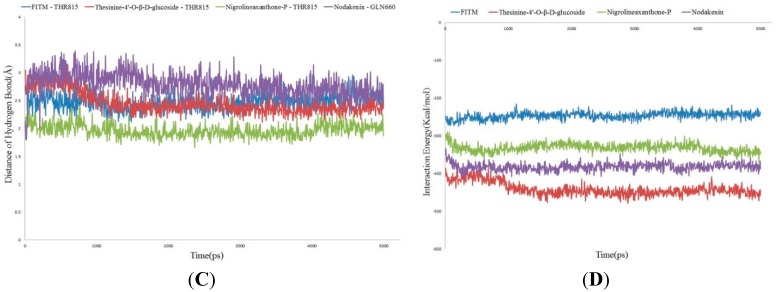
Results of the MD simulation of four complexes. (**A**) Average backbone RMSD; (**B**) Potential Energy; (**C**) Distance of hydrogen bond; (**D**) Interaction energy. Blue indicates FITM, red indicates thesinine-4ʹ-*O*-β-d-glucoside, green indicates nigrolineaxanthone-P, and purple indicates nodakenin.

Molecular dynamics (MD) simulation was conducted to evaluate stability of mGluR1-ligand complexes under dynamic conditions. The initial conformations of FITM and the three hits were acquired from the molecular docking experiments by LibDock. The RMSD curves of the receptor structures from each complex, potential energy profiles and interaction energy profiles of each complex are shown in Figure 6. The RMSD, potential energy and interaction energy of the models gets stabilized with the time, trajectories of complexes reached equilibrium after 4ns. The H-bond distances formed between FITM, thesinine-4ʹ-*O*-β-d-glucoside, nigrolineaxanthone-P and THR815 were within a range around 2.75, 2.50 and 2.00 Å and the distance trajectories also show that the H-bond of nodakenin with GLN660 averaged around 2.75 Å. THR815 was the key residue, which was identical with that reported in the literature [25,26]. The binding free energy values between ligands and mGluR1 were calculated. All three potential compounds have lower binding free energy values compared with the initial compounds. Taking all the evaluation indexes into consideration, those three compounds might have potential negative modulation effects on mGluR1.

## 3. Experimental Section

### 3.1. Data Collection and Preparation

By entering “metabotropic glutamate receptor 1” as a search term in the Allosteric Database [28], 85 mGluR1 NAMs were obtained. After removing all the compounds whose active values were not recorded, 74 mGluR1 NAMs were retained and then divided into three groups based on different structural types for further study [29,30,31]. The top six active molecules of each group were used as training sets for running GALAHAD models. Seventy four active compounds and 222 inactive compounds formed a test set to validate the selected pharmacophore models. In addition, 41 mGluR1 PAMs were used as external test set.

In case of pharmacophore modeling, the molecular geometry of each compound was minimized using the standard Tripos’ molecular mechanics force field. Partial atomic charges were added by the Gasteiger-Hückel method and the Conjugate Gradient method was used to perform the energy minimizations study with 1000 iterations.

For molecular docking, diverse conformations of all ligands were created by the BEST conformation generation method in Accelrys Discovery Studio 4.0 (DS) within the relative energy threshold of 20 kcal/mol [32]. Maximum number of conformations was set to 255. All other parameters were automatically set to the default value. 

### 3.2. Pharmacophore Model Studies

#### 3.2.1. GALAHAD Pharmacophore Hypotheses Generation

Genetic Algorithm with Linear Assignment of Hypermolecular Alignment of Datasets (GALAHAD) that is embedded in the SYBYL 7.0 package employs Tripos’ proprietary technology to generate pharmacophore hypotheses and alignments from sets of compounds that bind at a common target site.

There are two steps during GALAHAD operation. First, the compounds in the training set are aligned flexibly to each other in internal coordinate space. In this step, a genetic algorithm (GA) [33] was used to identify a set of conformations of compounds in the training set with minimized energy and maximized pharmacosteric similarity. Second, the conformations of the compounds produced are treated as rigid bodies and aligned in Cartesian space. This step makes use of linear assignment methodology and geometric heuristics to identify optimal feature correspondences between ligands [34]. Then, correspondences that are conserved are used to do a least-square alignment of the two ligands, and they are merged into a single hypermolecule. The procedure can then be applied to the hypermolecule produced and a third molecule, or to another hypermolecule. The end result of such an agglomerative process is a single master hypermolecule that incorporates information from each of the structures in the training set. Accordingly, the best models are carried forward into the second stage, multiplemodels are produced because a multi-objective fitness function is used in the GA, and every model represents a different trade-off among the competing criteria [35], but rarely do models achieve optimum values in all parameters. Additionally, it is important to add that the features which were considered during the development of models include hydrogen bond donor atoms, hydrogen bond acceptor atoms, hydrophobic and charged centers and so on.

#### 3.2.2. Validation of the Pharmacophore Model

The generated models were firstly evaluated by built-in parameters (Energy and Specificity). Besides, the top five models of each group would be selected and then evaluated by a test set using UNITY module. The test database composed of 74 experimentally known mGluR1 NAMs and 222 inactive compounds selected from DrugBank [36]. The evaluation indicators were presented as follows: Ha, Ht-Ha, A%, N, CAI [37,38]. Ha is the number of active hits using pharmacophores to search. Ht-Ha represents the number of false positive hits using pharmacophores to search. A% (the effectively hit ratio of active compounds) indicates the ability of pharmacophore model to identify active compounds from the test set. With a high value of A%, a pharmacophore model shows a strong ability to identify active compounds. N (namely the identified effective index) represents the ability to distinguish active compounds from inactive compounds. A hypothesis that possesses a high N value has a strong ability to distinguish active compounds from inactive ones. Then, Comprehensive Appraisal Index (CAI), which considers A% and N at the same time, is proposed to evaluate of the models comprehensively. In addition, 41 mGluR1 PAMs were using as external test set to further evaluate the identification ability of the optimal model. After considering every factor, the three best pharmacophore models for each structure type were utilized as queries to screen the compounds in the TCMD.

### 3.3. Database Search

The selected pharmacophore models were validated and converted into a UNITY query for pharmacophore-guided virtual screening studies. The “flexible database search” option was implemented to perform virtual screening. TCMD, which contains 233,033 natural compounds from 6735 medicinal plants, was the object of this screening. Moreover, the identified ligands were filtered by four rules of “Lipinski’s rule of five” in order to be further analyzed by the docking studies. The retained ligands must meet the following rules: MWT ≤ 500, LogP ≤ 5, H-bond donors ≤ 5, and H-bond acceptors ≤ 10 [39].

### 3.4. Molecular Docking Studies

#### 3.4.1. Define Binding Site

4OR2, a complex of the human mGluR1 receptor with a NAM in the RCSB Protein Data Bank, was used as a reference model. Common problems, such as incomplete residues, nonstandard atom order in amino acids, the lack of hydrogens and the existence of crystallographic waters, were automatically cleaned up by Prepare Protein protocol in DS. The protein-binding site was determined by combining results from experimental data and the Define and Edit Binding Site tools in DS. Meanwhile, all of the ligands used for the docking studies and the receptor 4OR2 were all assigned to their respective charge status corresponding to pH 7.0.

#### 3.4.2. Docking Strategy

LibDock, CDOCKER and flexible docking, three docking algorithms within DS, were used to evaluate their applicability for the binding site defining of 4OR2. After being extracted from the binding site, the initial compound FITM was re-docked into the crystal structure, and then, the RMSD was calculated. In general, an RMSD of less than 2.00 Å indicated it was highly reliable that a docking module would reproduce the experimentally observed binding mode for mGluR1 NAMs. The docking algorithm which obtained the smallest RMSD was selected for further utilizing. The molecular docking score of FITM was set to be the threshold in identifying mGluR1 NAMs.

In order to further analyze the different binding mode of three structure types of mGluR1 NAMs, active compounds in Group A, B and C were docked into the binding site, respectively. Then, the poses of the docked compounds were analyzed and the key residues of each structure type were obtained. Finally, potential compounds were selected on the basis of molecular docking score and favorable interaction with key residues.

### 3.5. Molecular Dynamics Simulation

Three potential NAMs of mGluR1 were chosen through the appraisal method mentioned above. The best binding conformation of them among the poses predicted by the molecular docking program was selected and the protein-NAM complexes were used as MD simulation starting points. In order to simulate the actual environment, implicit biological membranes were added to the protein-NAM complexes by using Generalized Born with Implicit Membrane (GBIM) model. Then, the system was subjected to the CHARMm force-field and relaxed by energy minimization (10,000 steps of steepest descent and 10,000 steps of conjugated gradient). The system was slowly driven from an initial temperature of 50 K to the target temperature of 300 K for 100 ps and equilibration simulations were run for 300 ps. The MD simulations (production) were performed for 600 ps with the NVT system at a constant temperature of 300 K and the results were saved at a frequency of 5000 ps. All the other parameters were set as defaults. The initial complex, protein-FITM, was set as a reference.

The MD trajectory was determined for structural properties, root mean-square deviation (RMSD), and potential energy by using the Discovery Studio 4.0 analyze trajectory protocol. The interaction energy between ligands and 4OR2 was then calculated using the calculate interaction energy protocol. All parameters were set as defaults.

## 4. Conclusions

In recent years, because of the decreased side effects, improved subtype selectivity and development of patient tolerance, allosteric modulator drug discovery has gradually gained great attention. Computational approaches, such as ligand-based pharmacophore modeling, molecular docking and structure-based homology modeling, are gaining importance as valuable methods to perform drug discovery. In this study, a series of computational methods were used to discovery potential mGluR1 NAMs from Traditional Chinese Medicines. To be specific, this work first attempted to build three pharmacophore models based on the different structure types of mGluR1 NAMs. The best pharmacophore model of each type which can distinguish NAMs not only from inactive compounds but also from PAMs is selected based on the validation with the test set and external test set. Then, the models were used for the TCMD virtual screening. Meanwhile, as the crystal structure of mGluR1-7TMD was obtained, detailed insight into the architecture of the transmembrane domains of class C GPCRs was provided, including the accurate location of the allosteric binding site and key residues that regulate receptor signaling. As a result, the development of allosteric modulator based on receptor structure is an effective method in this study. Thus, this paper represented the first successful attempt to use the resolved crystal structure of the mGluR1 to understand the ligand-receptor interactions and to reduce the false positive rate of ligand-based virtual screening. 30 potential mGluR1 NAMs were retained and the docking results suggested that several key residues (LEU757, ASN760, TRP798, PHE801, TYR805 and THR815) in mGluR1-7TMD played important roles in NAMs selectivity. Moreover, all the docking studies mentioned in our paper were consistent with the mutant experiments. Then, with the further evaluation by molecular dynamics studies, thesinine-4ʹ-*O*-β-d-glucoside, nigrolineaxanthone-P and nodakenin may serve as potential mGluR1 NAMs leads. The authors expect that the information gained from this study could be further employed in the search for potential natural mGluR1 NAMs.

The major shortcomings in this study are that some more complicated dynamic processes and the underlying mechanism of action of mGluR1 remain undiscovered because of the limitations of the current crystal structure. To be specific, firstly, the mechanism of allosteric modulators to modulate the affinity of orthosteric ligands is also unclear. Interestingly, however, as the orthosteric site and the allosteric site were both obtained in one crystal structure, there might be a deeper drug synergism contained in interactions of allosteric modulators and orthosteric compounds as described. A deeper drug synergism may be derived from the cooperativity of orthosteric and allosteric ligand binding which are electrostatic repulsion and the coupled conformations of the orthosteric and allosteric sites [40]. Secondly, although the NAMs screened by high specificity ligand-based pharmacophore models have been differentiated from PAMs, the binding modes of NAMs and PAMs under different conformational states are still unclear.

In the future, it is expected that other mGluR structures, such as active state structures in complex with PAMs or even a full length dimeric receptor structure, will be solved. Ultimately, leveraging the structural information from these mGluR receptors will allow computational approaches to further our understanding of class C GPCR activation mechanisms and promote the development process of allosteric modulators.
